# Past and present: a bibliometric study on the treatment of recurrent ovarian cancer

**DOI:** 10.3389/fphar.2024.1442022

**Published:** 2024-07-30

**Authors:** Xiao-yuan Hao, Wen-wei Song, Miao-ling Li, Yi Guo

**Affiliations:** ^1^ Department of Obstetrics and Gynecology, The Second Affiliated Hospital of Xuzhou Medical University, Xuzhou, Jiangsu, China; ^2^ Department of Laboratory Medicine, The Second Affiliated Hospital of Xuzhou Medical University, Xuzhou, Jiangsu, China; ^3^ Clinical Medical Research Center for Precision Medicine, The Second Affiliated Hospital of Xuzhou Medical University, Xuzhou, Jiangsu, China

**Keywords:** recurrent ovarian cancer, bibliometric analysis, platinum-based chemotherapy, angiogenesis inhibitors, poly (ADP-ribose) polymerase inhibitors

## Abstract

**Background:**

Ovarian cancer (OC) is a gynecological malignancy with a high mortality rate worldwide. The unfavorable prognosis of OC is mainly attributed to the recurrent propensity. Recently, mortality from OC has exhibited a downward trend. These favorable patterns are likely to be driven by advancements in novel therapeutic regimens. However, there is a lack of visualize analysis of the application of these new drugs on women with recurrent OC (ROC). Therefore, we aimed to provide a bibliometric analysis of the evolving paradigms in the ROC treatment.

**Methods:**

Documents on ROC treatment were systematically collected from the MEDLINE database and Web of Science Core Collection (WOSCC). The retrieved documents were exported in the plain text file format, and files were named and saved to the paths specified by the Java application. Microsoft Excel (version 2010), Citespace (6.2.R4) and VOSviewer (1.6.19) were used for data analysis, and included the following: 1) annual publication trend; 2) contributions of countries, institutions and authors; 3) co-citation of journals and references; and 4) co-occurrence of keywords.

**Results:**

A total of 914 documents published in the MEDLINE and 9,980 ones in WOSCC were retrieved. There has been an upward trend in the productivity of publications on ROC treatment on by years. The United States was the leading contributor in this field, and the University of Texas System stood out as the most productive institution. Giovanni Scambia and Maurie Markman were the research leaders in the field of ROC treatment. The journal *Gynecologic Oncology* had the highest citation frequency. The reference entitled with “Niraparib Maintenance Therapy in Platinum-Sensitive, Recurrent Ovarian Cancer” got highest centrality of 0.14 in the co-citation network. Keyword analysis revealed that the focus of current ROC treatment was on platinum-based anticancer drugs, paclitaxel, angiogenesis inhibitors (AIs), immune checkpoint inhibitors (ICIs) and poly (ADP-ribose) polymerase inhibitors (PARPis).

**Conclusion:**

Scholars from a multitude of countries have been instrumental in the advancement of ROC treatment. The research hotspots and trend in the field of predominantly originated from leading international journals and specialized periodicals focused on gynecologic oncology. Maintenance therapy using AIs or (and) PARPis has emerged as a significant complement to platinum-based chemotherapy for patients with ROC.

## 1 Introduction

Ovarian cancer (OC) is a gynecological malignancy with high mortality. In China, the crude and age-standardized death rates of OC have risen to 9.49/100,000 and 6.02/100,000, and it has become the leading cause of death in the female reproductive tract tumors (Zheng et al., 2023). The unfavorable prognosis of OC is mainly attributed to the advanced disease stage detection and recurrent propensity. The standard therapeutic regimen for patients with advanced ovarian cancer is cytoreductive surgery followed by platinum-based chemotherapy (Moore et al., 2018). Surgical cytoreduction of advanced stage ovarian cancer, also termed “tumor debulking,” is defined as an attempt to maximally resect all visible and palpable disease. The procedure includes, but is not limited to, hysterectomy and salpingo-oophorectomy, peritonectomy with or without gastrointestinal surgery, lymph node dissection, omentectomy and upper abdominal surgery (Polcher et al., 2014). Due to the underestimated incidence of hepatobiliary involvement in advanced OC, diaphragms and porta hepatis should be also explored during cytoreductive surgery to identify potentially undetected disease at preoperative instrumental examinations (Di Donato et al., 2021). The Gynecologic Oncology Group defined a maximum tumor diameter of 1 cm or less as an “optimal debulking” status. Approximately 70% of patient will have a relapse within the subsequent few years, despite a complete response to the optimal debulking surgery accompanied by chemotherapy (Richardson et al., 2023). Recurrent ovarian cancer (ROC) is rarely curable, with most patients receiving multiple additional lines of treatment before ultimately dying from the disease (Moore et al., 2018). The dismal destiny of patients with ROC has changed little over the past three decades.

Nevertheless, mortality of ROC has exhibited a downward trend in recent years, especially in the western countries. Accelerated declines of ROC mortality could be observed from 2017 to 2020 (Siegel et al., 2023). The age-standardize death rate of ROC fell by 6% in 2022, reaching 4.3 deaths per 100,000 individuals. This decline is predicted to continue until at least 2025 (Dalmartello et al., 2022; Wojtyła et al., 2023). These favorable patterns likely find their main driving factors for advancements in novel therapeutic regimens (Wojtyła et al., 2023). It is therefore necessary to identify the new drugs that work and to understand their evolving paradigms in the treatment of ROC. Compared to the narrative reviews, the bibliometric review could comprehensively include related studies and provide quantitative results/In comparison to narrative reviews, bibliometric reviews have the capacity to encompass a wide range of relevant studies, and present quantitative and visualized findings in a comprehensive manner (Cai et al., 2023). Therefore, in this study, we aim to perform a bibliometrics analysis to present the evolution and current status of ROC treatment, providing researchers with hotspots and frontiers in the field.

## 2 Methods

### 2.1 Data retrieval

We systematically searched for the documents about ROC treatment in the MEDLINE database via the Pubmed website (https://pubmed.ncbi.nlm.nih.gov/) and Web of Science Core Collection (WOSCC) (https://www.webofscience.com/wos/woscc/basic-search). The retrieved publications were required to meet the inclusion criteria: 1) the search terms were determined by the TS (“topic,” including title, abstract, and keywords) as TS = (“ovarian cancer*” OR “ovarian neoplasm*” OR “ovarian carcinoma”) AND TS = (“recurren*” OR “relapse*”) AND TS = (“therap*” OR “treatment*” OR “management”); 2) the period of publication spanned from 1960 to 2023; 3) the article language was limited to English; 4) the following information should be found: publication, authors, countries, institutions, journals, keywords, and citations. The literature obtained was screened based on the following exclusion criteria: publications unrelated to the topic, articles not officially published, meeting summary, repeated articles and incomplete articles. Two authors (Wen-wei Song and Miao-ling Li) independently conducted the data retrieval. Discrepancies were solved through discussion, and when needed a third researcher (Yi Guo) was consulted. Ethics approval and consent to participate were not applicable for the study, since we retrospectively searched the data from public databases.

### 2.2 Data export

The retrieved documents were exported in the format of plain text file. One file comprised 500 records, and each record included author (s), title, publication year, document source, abstract, addresses, affiliations, document type, keywords, cited references, and total citations. Files were named and saved to the paths specified by the Java application.

### 2.3 Data analysis

Microsoft Excel (version 2010), Citespace (6.2.R4) and VOSviewer (1.6.19) were used for data analysis. We recorded the numbers of published documents yearly and presented the annual publication trend via Microsoft Excel. Citespace was utilized to evaluate the contributions of countries, institutions and authors to the ROC treatment, as well as the co-citation of journals and references. The co-occurrence of keywords in the field was depicted in the forms of cluster analysis, hotspot distribution and evolution tendency by VOSviewer.

## 3 Results

### 3.1 Annual publication trends

**FIGURE 1:**
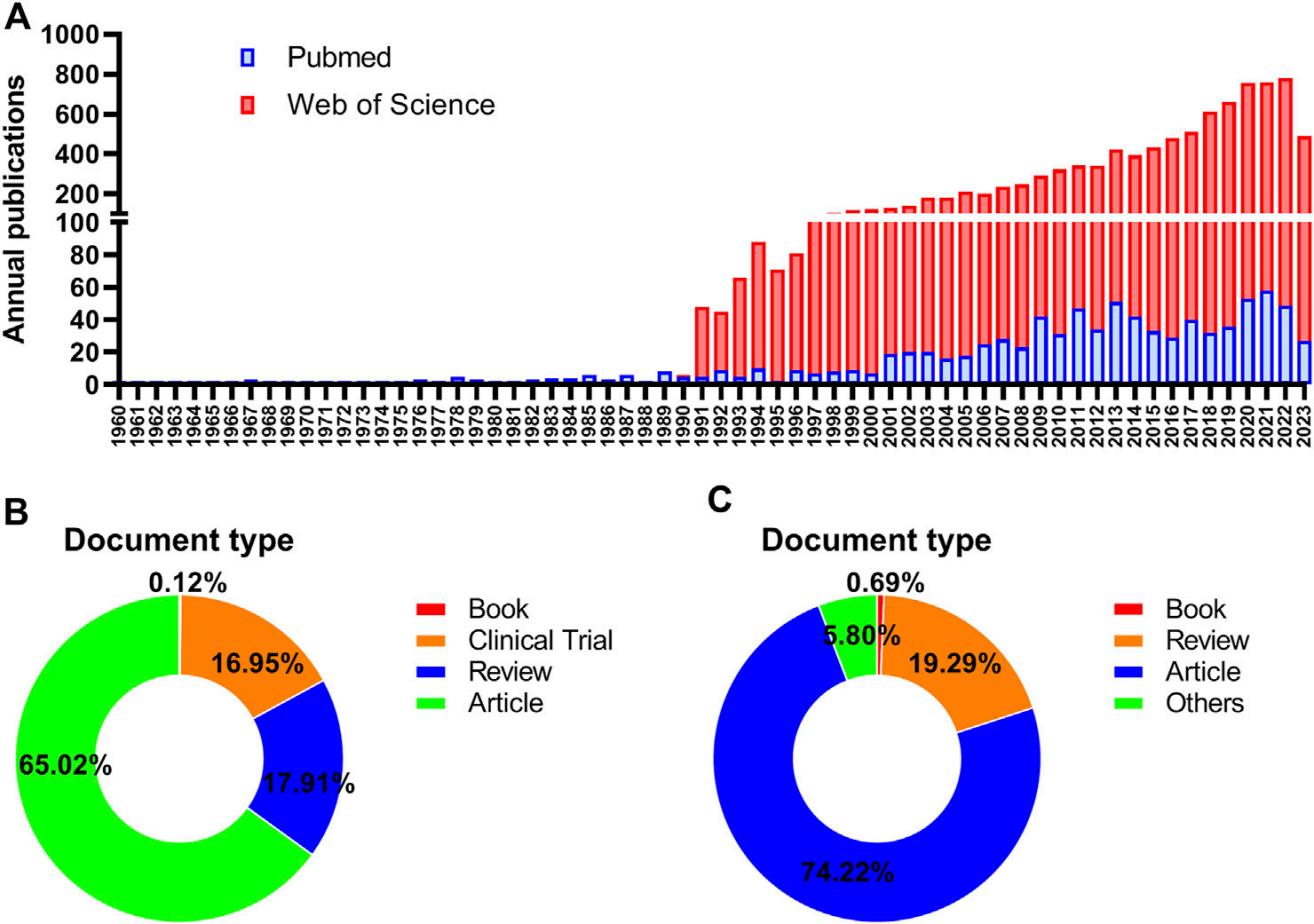
Annual publications in the field of ROC treatment **(A)** Annual number trend of publications about recurrent ovarian cancer and therapy in the Pubmed and Web of Science database **(B)** The distribution of document type in the Pubmed database **(C)** The distribution of document type in the Web of Science database. Note: ROC: recurrent ovarian cancer.

In the light of our search strategies, a total of 914 documents pertaining to ROC treatment were collected in the MEDLINE database database spanning the years 1960–2023, while 9,980 ones were retrieved in the WOSCC database for the period between 1977 and 2023. Figure 1A showed the distribution of the related documents over the past few decades. Generally, there has been an upward trend in the productivity of publications by years. The ascent process exhibited two distinct phases: a period of rapid growth from 1990 to 1999, followed by a period of consistent increase from 2000 to 2022. The trend indicated that researches on the ROC treatment looked to usher a favorable turn after a period of frustration. In terms of the document type, original articles accounted for above two-thirds (65.02%) in the MEDLINE and almost three-quarters (74.22%) in the WOSCC. The proportion of other types could be seen in the Figures 1B, C.

### 3.2 The contributions of countries/regions in the research of ROC treatment

**FIGURE 2:**
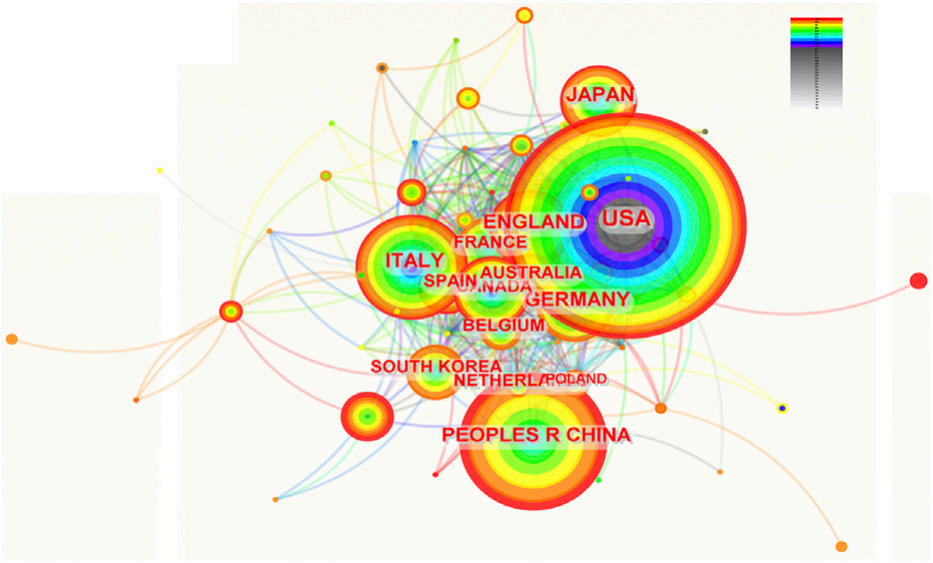
The network of countries and institutions involved in ROC treatment. Notes: ROC, recurrent ovarian cancer. Each node represents each country. The size of nodes represents the number of publications. The color of the layer of nodes represents the year of publication. The connection between nodes represents the cooperation between countries. The color of the connecting line represents the cooperation time.

Scholars from 66 countries/regions have authored at least one academic paper pertaining to ROC. Figure 2 depicted the contributions of these countries and the connections among them. The top 10 countries ranked by the number of publications were the United States, China, Italy, England, Germany, Japan, France, Canada, Australia and Spain (Table 1). It is worth noting that the developed countries have made the major contributions to the publications, though China ranked second with 1,120 records. In addition, the United States and Italy achieved the highest centrality (0.08), followed by France and Australia (centrality = 0.06) (Table 1). These nations were instrumental in advancing research in this field and were seen as conduits for disseminating the innovative ethos to other regions.

**TABLE 1:** The top 10 countries contributing to the research of ROC treatment.

Rank	Country	Counts	Centrality	Year
1	The United States	3,499	0.08	1993
2	China	1,120	0.01	2000
3	Italy	1,065	0.08	1993
4	England	785	0.04	1993
5	Germany	785	0.02	1993
6	Japan	677	0.01	1993
7	France	588	0.06	1993
8	Canada	568	0.04	1993
9	Australia	402	0.06	1993
10	Spain	392	0.01	1997

ROC, recurrent ovarian cancer.

### 3.3 The contributions of institutions in the research of ROC treatment

**FIGURE 3:**
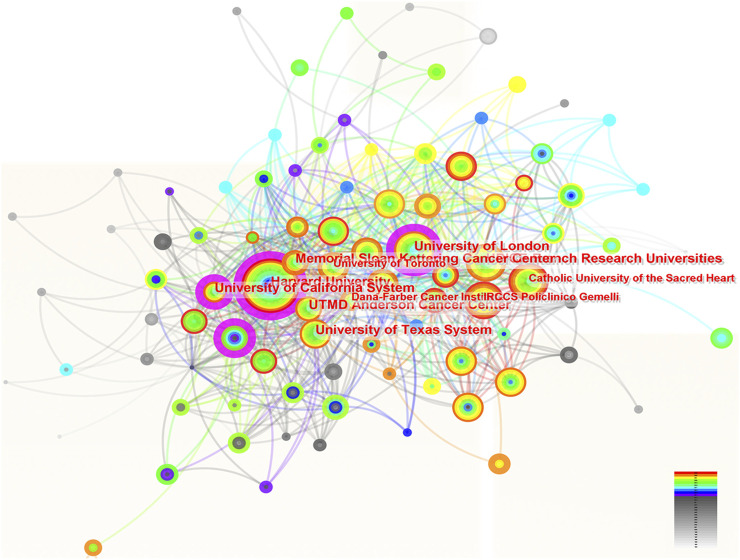
The network of institutions involved in ROC treatment. Notes: ROC, recurrent ovarian cancer. Each node represents each institution. The size of nodes represents the number of publications. The color of the layer of nodes represents the year of publication. The connection between nodes represents the cooperation between institutions. The color of the connecting line represents the cooperation time.

A total of 109 institutions were involved in the research of ROC treatment independently or by collaboration. Figure 3 portrayed the contributions of these organizations and the relations between each other. The top 10 institutions listed by the productivity of publications were University of Texas System, UT MD Anderson Cancer Center, Harvard University, French Research Universities (UDICE), University of California System, Memorial Sloan Kettering Cancer Center, University of London, Catholic University of the Sacred Heart, University of Toronto and Dana-Farber Cancer Institute (Table 2). Among the top 10 organizations, six were from United States, which reflected its great scientific strength in this area. However, the institution with highest centrality (0.13) was University of London from England, followed by University of California System (centrality = 0.12) and Harvard University (centrality = 0.11) (Table 2).

**TABLE 2:** The top 10 institutions contributing to the research of ROC treatment.

Rank	Institution	Counts	Centrality	Year
1	University of Texas System	506	0.09	1994
2	UT MD Anderson Cancer Center	435	0.09	1995
3	Harvard University	426	0.11	1994
4	French Research Universities (UDICE)	302	0.08	1996
5	University of California System	280	0.12	1994
6	Memorial Sloan Kettering Cancer Center	271	0.08	1993
7	University of London	254	0.13	2004
8	Catholic University of the Sacred Heart	241	0.04	2003
9	University of Toronto	228	0.06	2000
10	Dana-Farber Cancer Institute	201	0.08	2001

ROC, recurrent ovarian cancer.

### 3.4 The contributions of authors in the research of ROC treatment

**FIGURE 4:**
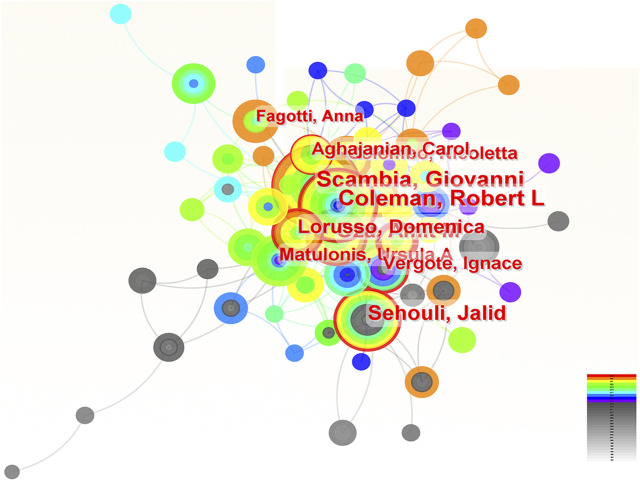
The analysis of authors dedicated to ROC treatment. Notes: ROC, recurrent ovarian cancer. Each node represents each author. The size of nodes represents the number of published documents. The color of the layer of nodes represents the year of publication. The connection between nodes represents the cooperation between authors. The color of the connecting line represents the cooperation time.

The number of authors with more than two papers in the field of ROC treatment was 162. The contributions of these authors and the pattern of interactions among them were delineated in the Figure 4. The top 10 authors with the most amounts of publications were Giovanni Scambia, Robert L Coleman, Jalid Sehouli, Amit M Oza, Domenica Lorusso, Ursula A Matulonis, Nicoletta Colombo, Carol Aghajanian, Ignace Vergote and Anna Fagotti (Table 3). Among them, four were from Italy, three from the United States, one from Germany, one from Canada and one from Belgium. Researchers from developed countries were the backbone in the field, and Robert L Coleman from the United States occupied the core position in the network (centrality = 0.07), followed by Giovanni Scambia from Italy (centrality = 0.06) and Ursula A Matulonis from the United States (centrality = 0.05) (Table 3).

**TABLE 3:** The top 10 authors contributing to the research of ROC treatment.

Rank	Author	Count	Centrality	Year
1	Giovanni Scambia	119	0.06	2011
2	Robert L Coleman	110	0.07	2010
3	Jalid Sehouli	84	0.03	2008
4	Amit M Oza	65	0.02	2015
5	Domenica Lorusso	51	0.01	2017
6	Ursula A Matulonis	45	0.05	2012
7	Nicoletta Colombo	42	0.03	2015
8	Carol Aghajanian	38	0.02	2015
9	Ignace Vergote	35	0.02	2013
10	Anna Fagotti	32	0.00	2018

ROC, recurrent ovarian cancer.

### 3.5 The analysis of co-cited authors in the field of ROC treatment

**FIGURE 5:**
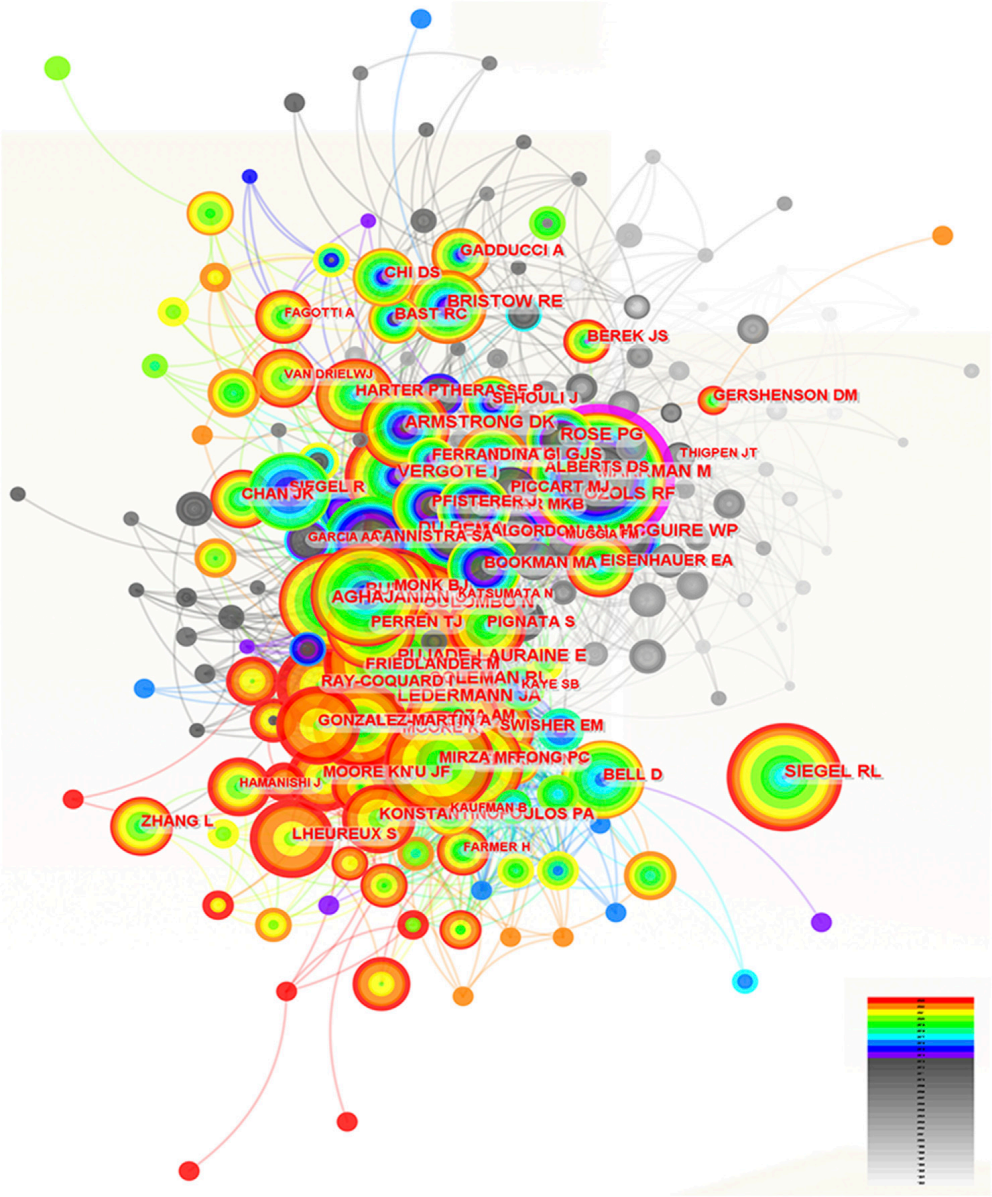
The analysis of co-cited authors dedicated to ROC treatment. Notes: ROC, recurrent ovarian cancer. Each node represents each author. The size of nodes represents the number of published documents. The color of the layer of nodes represents the year of publication. The connection between nodes represents the cooperation between authors. The color of the connecting line represents the cooperation time.

In total, 253 authors were co-cited by multiple articles due to their excellent research achievements in the field of ROC treatment. The pattern of citation for these authors and their cooperation were showed in the Figure 5. The top 10 authors with the most co-citations were Maurie Markman, Eric Pujade-Lauraine, Robert F Ozols, Andreas du Bois, Robert L Coleman, Robert A Burger, Rebecca L Siegel, Ignace Vergote, William P McGuire and Nicoletta Colombo (Table 4). They were all from developed countries, and six of them were from the United States. Research findings from Maurie Markman were well recognized and widely cited by experts in the field, so he got the highest centrality (0.12) (Table 4). Andreas du Bois and Robert A Burger were tied for second (centrality = 0.08), and Ignace Vergote ranked third (centrality = 0.06) (Table 4).

**TABLE 4:** The top 10 co-cited authors in the field of ROC treatment.

Rank	Co-cited author	Count	Centrality	Year
1	Maurie Markman	1,540	0.12	1993
2	Eric Pujade-Lauraine	1,221	0.05	2011
3	Robert F Ozols	1,006	0.04	1993
4	Andreas du Bois	975	0.08	2004
5	Robert L Coleman	967	0.05	2013
6	Robert A Burger	930	0.08	2006
7	Rebecca L Siegel	870	0.00	2010
8	Ignace Vergote	830	0.06	2001
9	William P McGuire	817	0.03	1993
10	Nicoletta Colombo	741	0.02	1999

ROC, recurrent ovarian cancer.

### 3.6 The analysis of co-cited journals in the field of ROC treatment

**FIGURE 6:**
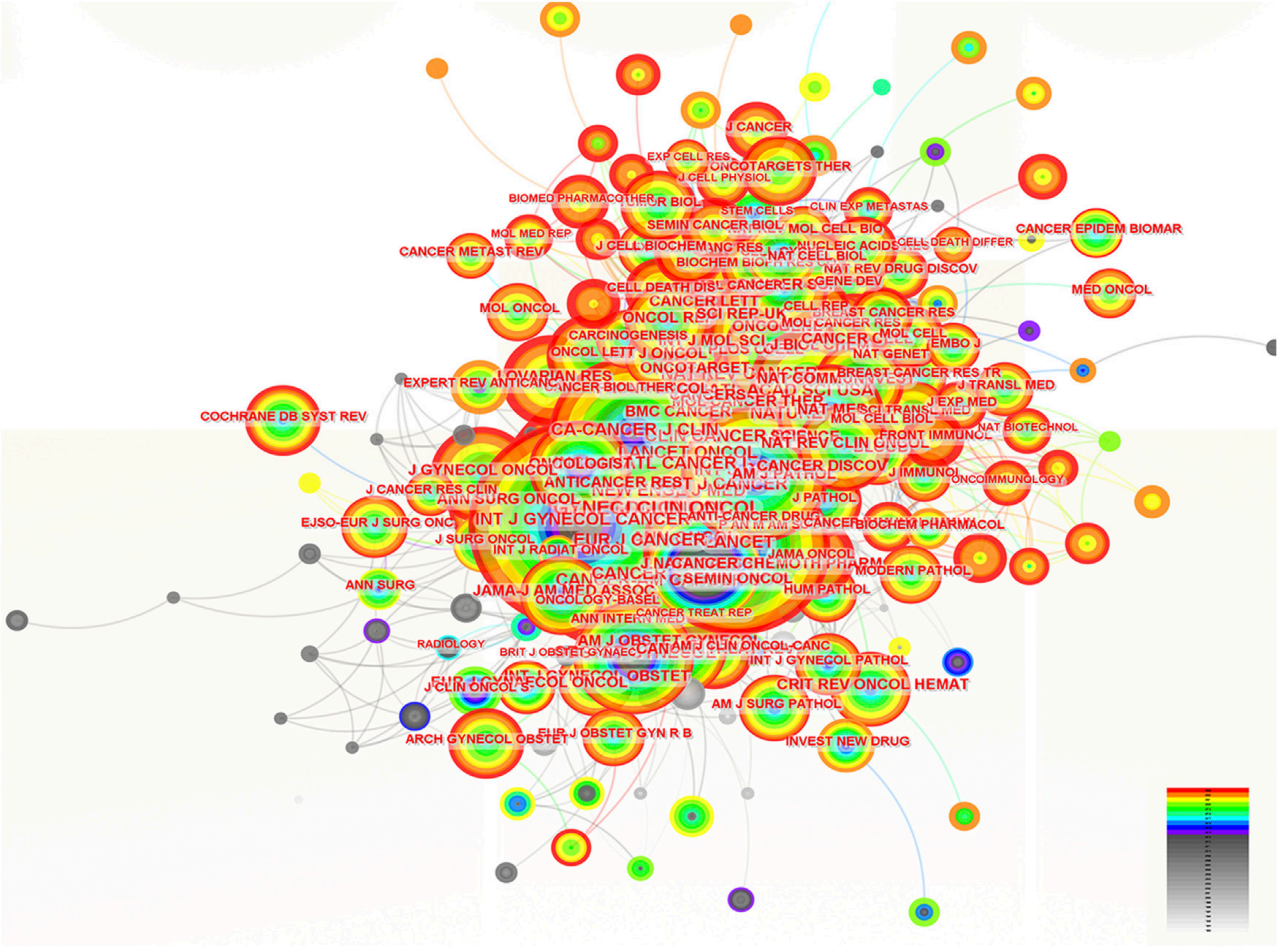
The analysis of co-cited journals related to ROC treatment. Notes: ROC, recurrent ovarian cancer. Each node represents each journal. The size of nodes represents the number of published documents. The color of the layer of nodes represents the year of publication.

Two hundred and eighty-three journals were co-cited by the literature on ROC treatment. The number of these journals cited and when cited can be seen in Figure 6. The top 10 journals ordered by frequency of citation were the *Gynecologic Oncology*, the *Journal of Clinical Oncology*, the *New England Journal of Medicine*, the *Cancer Research*, the *Annals of Oncology*, the *Clinical Cancer Research*, the *International Journal of Gynecological Cancer*, the *British Journal of Cancer*, the *Lancet* and the *Cancer* (Table 5). Two of the 10 journals fell into OBSTETRICS and GYNECOLOGY category, seven belonged to ONCOLOGY category and two were in the category of GENERAL MEDICINE. In addition, there were two journals with impact factors above 100.0 (the *New England Journal of Medicine* and the *Lancet*), 4 with impact factors between 10.0 and 100.0 (the *Annals of Oncology*, the *Journal of Clinical Oncology*, the *Clinical Cancer Research* and the *Cancer Research*) and 4 with impact factors below 10.0 (the *Gynecologic Oncology*, the *International Journal of Gynecological Cancer*, the *British Journal of Cancer* and the *Cancer*). Details could be seen in the Table 6. Remarkably, the focal point journal was the *Cancer Research* with a centrality score of 0.12, followed by the *Gynecological Cancer* (centrality = 0.06) and *England Journal of Medicine* (centrality = 0.04) (Table 5).

**TABLE 5:** The top 10 co-cited journals in the field of ROC treatment.

Rank	Co-cited journal	Count	Centrality	Year
1	Gynecologic Oncology	6,927	0.06	1993
2	Journal of Clinical Oncology	6,579	0.03	1993
3	New England Journal of Medicine	4,606	0.04	1993
4	Cancer Research	4,007	0.12	1993
5	Annals of Oncology	3,974	0.03	1993
6	Clinical Cancer Research	3,859	0.02	1997
7	International Journal of Gynecolo gical Cancer	3,812	0.01	1998
8	British Journal of Cancer	3,543	0.03	1993
9	Lancet	3,021	0.01	1993
10	Cancer	2,995	0.02	1993

ROC, recurrent ovarian cancer.

**TABLE 6:** The impact factors of the top 10 co-cited journals in the field of ROC.

Rank	Co-cited journal	IF 2022–2023	IF 5 years
1	Gynecologic Oncology	4.7	5.0
2	Journal of Clinical Oncology	45.3	37.6
3	New England Journal of Medicine	158.5	115.7
4	Cancer Research	11.2	13.0
5	Annals of Oncology	50.5	32.4
6	Clinical Cancer Research	11.5	12.5
7	International Journal of Gynecological Cancer	4.8	4.0
8	British Journal of Cancer	8.8	8.4
9	Lancet	168.9	118.1
10	Cancer	6.2	6.8

ROC, recurrent ovarian cancer; IF, impact factors.

### 3.7 The analysis of co-cited references in the field of ROC treatment

**FIGURE 7:**
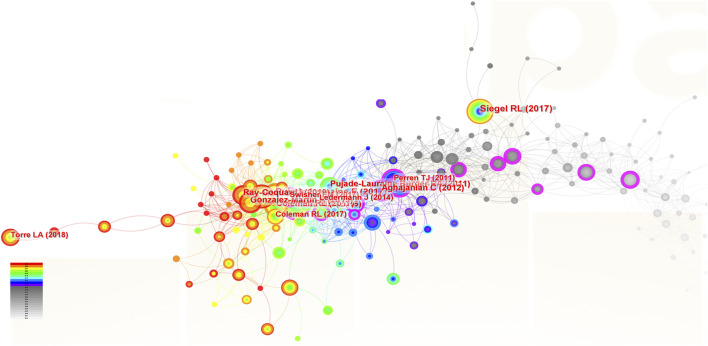
The analysis of co-cited references related to ROC treatment. Notes: ROC, recurrent ovarian cancer. Each node represents each reference. The size of nodes represents the number of published documents. The color of the layer of nodes represents the year of publication.

Two hundred and fifteen papers were identified and cited as references in the studies focus on the ROC therapy. In Figure 7, the size of the nodes corresponded to the frequency of citation, the color layer of the nodes signified the year of citation, and the links connecting the nodes indicated that the two references were cited by the same paper. The top 10 references with most citations were listed in the Table 7. Among them, five were published on the *New England Journal of Medicine*, two were on the *Lancet*, two were on the *CA-A Cancer Journal for Clinicians* and one were on the *Journal of Clinical Oncology*. The publication dates of the 10 most cited references spanned from 2011 to 2020. The themes and subjects of these references mainly centered on maintenance therapy based on the poly (ADP-ribose) polymerase inhibitors (PARPis) (e.g., olaparib, niraparib and rucaparib), the angiogenesis inhibitors (AIs) (e.g., bevacizumab), and cancer statistics. The clinical trial titled “Niraparib Maintenance Therapy in Platinum-Sensitive, Recurrent Ovarian Cancer” by (Mirza et al., 2016) published in the *New England Journal of Medicine* in 2016 held a prominent position in the co-citation network with a centrality score of 0.14.

**TABLE 7:** The top 10 co-cited references in the field of ROC.

Rank	Title	Count	Centrality	Journal	Year	First author
1	Cancer statistics, 2017	571	0.05	CA-A Cancer Journal for Clinicians	2017	Rebecca L Siegel
2	Maintenance olaparib in patients with newly diagnosed advanced ovarian cancer	421	0.04	New England Journal of Medicine	2018	Kathleen Moore
3	Olaparib tablets as maintenance therapy in patients with platinum-sensitive, relapsed ovarian cancer and a BRCA1/2 mutation (SOLO2/ENGOT-Ov21): a double-blind, randomised, placebo-controlled, phase 3 trial	421	0.08	Lancet	2017	Eric Pujade-Lauraine
4	Niraparib maintenance therapy in platinum-sensitive, recurrent ovarian cancer	382	0.14	New England Journal of Medicine	2016	Mansoor R Mirza
5	Rucaparib maintenance treatment for recurrent ovarian carcinoma after response to platinum therapy (ARIEL3): a randomised, double-blind, placebo-controlled, phase 3 trial	368	0.05	Lancet	2017	Robert L Coleman
6	Niraparib in patients with newly diagnosed advanced ovarian cancer	312	0.03	New England Journal of Medicine	2019	Antonio González-Martín
7	Olaparib plus bevacizumab as first-line maintenance in ovarian cancer	286	0.02	New England Journal of Medicine	2020	Isabelle Ray-Coquard
8	Bevacizumab combined with chemotherapy for platinum-resistant recurrent ovarian cancer: the aurelia open-label randomized phase III trial	286	0.10	Journal of Clinical Oncology	2014	Eric Pujade-Lauraine
9	Global cancer statistics 2018: GLOBOCAN estimates of incidence and mortality worldwide for 36 cancers in 185 countries	252	0.00	CA-A Cancer Journal for Clinicians	2018	Freddie Bray
10	Incorporation of bevacizumab in the primary treatment of ovarian cancer	251	0.13	New England Journal of Medicine	2011	Robert A Burger

ROC, recurrent ovarian cancer.

### 3.8 The analysis of co-occurrence keywords about ROC treatment

**FIGURE 8:**
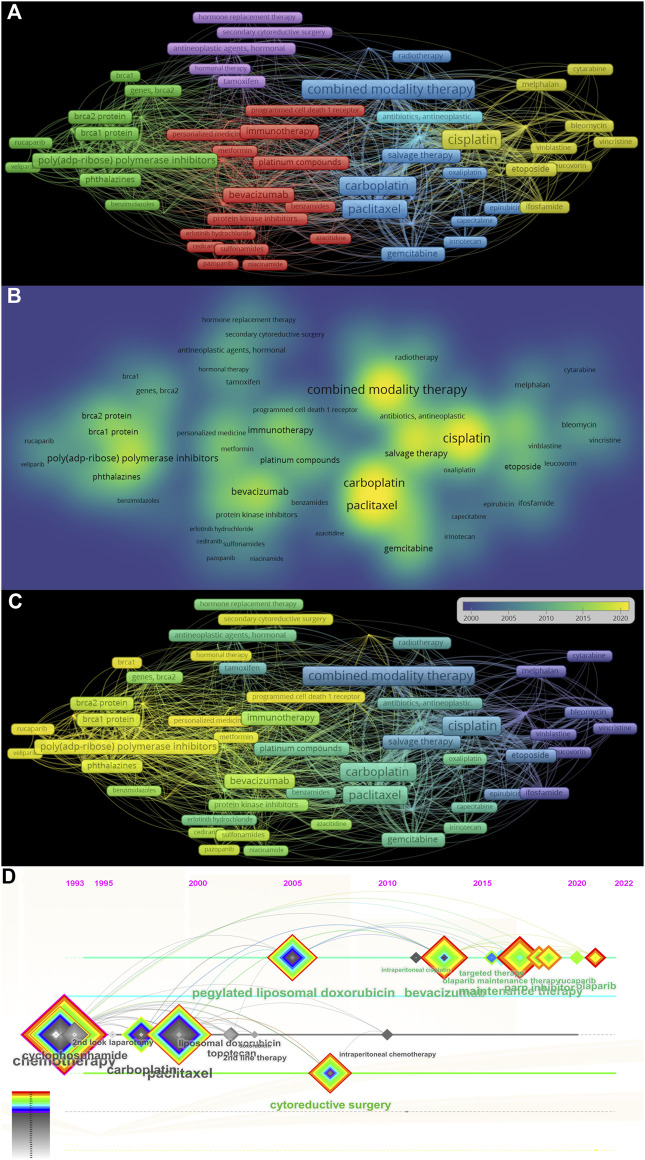
The analysis of co-occurrence keywords related to ROC treatment **(A)** The cluster view of co-occurrence keywords in the researches regarding recurrent ovarian cancer treatment **(B)** The density map of co-occurrence keywords **(C)** The evolution of the co-occurrence keywords **(D)** The timeline view of co-occurrence keywords. Note: ROC, recurrent ovarian cancer. The more frequently the keyword co-occur, its background color is closer to yellow in the **(B)**.

We totally got 2,777 terms related to the treatment of ROC based on the minimum number of occurrences (10) and the relevance score (60%). In order to remove general terminology and categorize specific terms, a cluster analysis was performed. The specific keywords were sorted into five clusters, as showed in the Figure 8A. The yellow cluster represented combination therapy strategies utilizing the first-generation platinum-containing anticancer drug (cisplatin), the blue cluster served as combined modality therapy involving the second- and third-generation platinum-based chemotherapeutic agents (carboplatin and oxaliplatin) and paclitaxel, the purple cluster mainly meant the hormonal treatment, such as tamoxifen, the red cluster primarily spoke of the induction of immunotherapy, including anti-PD-1, AIs (bevacizumab) and anti-protein kinase receptors (cediranib and pazopanib), and the green cluster stood for the maintenance therapy based on PARPis (niraparib and rucaparib). The density map in Figure 8B indicated that while targeted therapy has gained increasing attention, cytotoxic drugs such as platinum agents and paclitaxel remain essential for the treatment of ROC. The overlay visualization in Figure 8C depicted the evolution trends of keywords in this area over time, suggesting the transitions from cytotoxic agents to targeted therapy drugs. From the timeline view in the Figure 8D, we found that bevacizumab and PARPis have gained popularity in the years 2010 and 2016, respectively. However, their close links with cytotoxic drugs implied the continued value of classical chemotherapy in the treatment of ROC.

## 4 Discussion

### 4.1 Main findings of the study

This study represents the first bibliometric analysis to investigate the evolution in the treatment of ROC from the 1960 to 2023. In the study, we visualized current global research landscape on ROC therapy from multiple perspectives, such as involved researchers, countries, institutions, co-cited journals and co-cited keywords. We expected that these findings could offer valuable information for therapeutic decision-making in ROC, and the principal findings included the following:

(1) Research on the treatment of ROC has shown a consistent upward trend in recent years, presenting the global challenge posed by ROC and effort from worldwide to combat this disease.
(2) Scholars and institutions from developed countries like the United States and Italy have made significant contributions in helping OC patients fight against recurrence; China, the sole developing country on the list, required increased cooperation with other countries.
(3) The co-cited journals in the field of ROC treatment predominantly consisted of prominent international journals and specialized periodicals dedicated to the study of gynecological oncology.
(4) The co-cited references primarily focused on assessing the efficacy of bevacizumab and PARPis as monotherapy or in combination on patients with ROC and newly diagnosed OC.
(5) While chemotherapy still occupied an important position in the treatment of ROC, targeted therapeutic agents like AIs, ICIs and PARPis have emerged as research hotspots and publication trends; traditional chemotherapy and targeted therapy have been closely linked in the field of ROC treatment.

### 4.2 Implications, comparison with literature and future directions

#### 4.2.1 General information

Based on the annual publication trends, we found that research on ROC treatment has steadily increased over the years and is projected to continue growing in 2023. Liu et al. (2023) also observed the upward trend in the number of publications in the past decade, but only in the field of OC and drug resistance. The first surge of studies in the field likely commenced in 1994, following the approval of paclitaxel was approved for the treatment of ROC by the United States Food and Drug Administration (FDA) (Menzin et al., 1994). Since then, numerous trials have been conducted to assess the efficacy of paclitaxel as salvage chemotherapy in patients with platinum-sensitive EOC and those with platinum-resistant disease (Christian and Trimble, 1994; Miglietta et al., 1997; Roland et al., 1998). Meanwhile, more agents like 5-fluorouracil, leucovorin, merbarone and tamoxifen, were being assessed in clinical trials; however, only a small subset of the patients gained benefits (Look et al., 1992; Look et al., 1996; Trope et al., 2000), transiently impeding the progression of the research in this field. It was not until the year 2005 that the growth in the number of publications began to resume. Pfisterer et al. (2005) demonstrated that gemcitabine significantly prolonged progression-free survival (PFS) of patients with platinum-sensitive recurrence (PSR) when used as a second-line combination therapy, which prompted the next accelerated approval by the United States FDA in 2006 (Shea et al., 2013). Subsequently, the availability of targeted therapy for solid tumors like lung, breast, colorectal and renal cancers encouraged gynecological oncologists to incorporate these non-cytotoxic agents into ROC regimen (Palazzo et al., 2010). Therefore, related publications from 2010 to 2020 have been characterized as a steady upward curve, during which the targeted therapy has ushered a new era for ROC treatment.

#### 4.2.2 Contributions of the countries, the institutions, and the authors

Dozens of countries have dedicated significant effort and resources to improve ROC treatment, underscoring the global challenge posed by managing patients with ROC. The Gynecologic Cancer InterGroup (GCIG) consists of thirty-three clinical research groups that span the globe, and has organized an ovarian cancer consensus conference on clinical research including recurrent disease approximately every 5 years (Vergote et al., 2022). The East Asian Gynecologic Oncology Trial Group (EAGOT) was create to optimize ROC treatment across Japan, Korea, China and Taiwan (Kobayashi et al., 2024). Among them, the United States has become the leading stronghold to help ovarian cancer patients against recurrence: it contributed to the most publications with highest betweenness centrality; six of the top 10 institutions engaged in research on ROC were located in the United States; three authors and six co-cited authors in the ranking lists were American. Italy ranked three among the top 10 countries with the highest number of publications, with the same centrality score as the United States. These results are consistent with the systematic reviews, which have reported that researchers from both the United States and Italy have been actively involved in the majority of significant clinical trials that inform treatment protocols for ROC (Liu et al., 2022; Li et al., 2023). China was the sole developing country on the list, attributed to the government’s recognition of the escalating annual mortality rates of ovarian cancer (Feng et al., 2023), leading to increased funding and research efforts in this area. The phase III NORA study has been conducted funded by the National Major Scientific and Technological Special Project for Significant New Drugs Development (grant number: 2018ZX09736019) to evaluate the efficacy and safety of niraparib for the treatment of Chinese patients with platinum-sensitive ROC (Wu et al., 2021). However, the centrality score of China was low, indicating the urgent need for collaboration. Indeed, clinical trials conducted by Chinese scholars mainly were single-center studies (Ni et al., 2021; Wang et al., 2023). Other developed countries were centered on the United States and Italy, and work closely together. For instance, Amit M Oza from Canada has participated in the ARIEL3 study conducted by Giovanni Scambia (Italy) and Robert L Coleman (the United States) to evaluate the efficacy of rucaparib maintenance treatment for ROC (Coleman et al., 2017).

#### 4.2.3 Analysis of co-cited journals and co-cited references

By analyzing the co-cited journals and co-cited references with high frequency, we could gain an insight into the source of the research trends and highlights within the field. This study identified the top ten most frequently co-cited sources.

Two of the top ten co-cited journals concerned gynecologic tumors (the *Gynecologic Oncology* and the *International Journal of Gynecological Cancer*), six were journals in cancer research and oncology (the *Journal of Clinical Oncology*, the *Cancer Research*, the *Annals of Oncology*, the *Clinical Cancer Research*, the *British Journal of Cancer* and the *Cancer*), and two were comprehensive medical periodicals (the *New England Journal of Medicine* and the *Lancet*). Similarly, Duan et al. (2023) found that the *Gynecologic Oncology* published the most papers about platinum-resistant ovarian cancer research, and the *Journal of Clinical Oncology* received the largest number of co-citations; The *New England Journal of Medicine of Medicine* published numerous studies highlighting significant advancements in the field of oncology (Tu et al., 2022). To be specific, the *New England Journal of Medicine* and the *Lancet* are renowned for publishing top-notch medical research, the *Journal of Clinical Oncology* and the *Annals of Oncology* concentrate on clinical trials evaluating the effectiveness of different anti-cancer medications, the *Clinical Cancer Research* and the *British Journal of Cancer* publish translational cancer research studies that bridge the laboratory and the clinic, the *Cancer Research* and the *Cancer* provide oncological studies on basic, clinical and epidemiological research, the *Gynecologic Oncology* and *International Journal of Gynecological Cancer* are devoted to the publications for topics relevant to the etiology, mechanism, diagnosis, and treatment of gynecologic malignancies. In addition, the journal with the highest impact factor (IF) in 2022–2023 is the *Lancet* (168.9), followed by the *New England Journal of Medicine* (IF = 158.5). There are two journals with IF > 40.0 (the *Annals of Oncology* and the *Journal of Clinical Oncology*), two with IF > 10.0 (the *Cancer Research* and the *Clinical Cancer Research*) and two with IF > 5.0 (the *British Journal of Cancer* and the *Cancer*). For the left two, the *Gynecologic Oncology* is the official publication of the Society of Gynecologic Oncology with the second highest centrality scores, and the *International Journal of Gynecological Cancer* is the official journal of the International Gynecologic Cancer Society and the European Society of Gynecological Oncology. These data indicated that the research hotspots in the field of ROC treatment predominantly originated from leading international journals and specialized periodicals focused on gynecologic oncology.

The references that ranked first and ninth pertained to statistical analysis of global cancer incidence and mortality. Given their relevance to the epidemiological characteristics of ovarian cancer, they were deemed essential for citation in the background section of each manuscript. The earliest published paper among the top 10 co-cited references was titled with “Incorporation of Bevacizumab in the Primary Treatment of Ovarian Cancer” issued on the *New England Journal of Medicine* in 2011. In this study, Burger et al. (2011) integrated bevacizumab into the standard front-line therapy and observed that the combination extended the median progression-free survival by approximately 4 months in patients with newly diagnosed advanced ovarian cancer. Three years later, Pujade-Lauraine et al. (2014) presented evidence from their AURELIA study demonstrating that bevacizumab enhanced the efficacy of chemotherapy for OC patients with platinum-resistant recurrence, which has received 286 citations according to our statistical analysis. These data have facilitated the approval of bevacizumab for the management of OC in 2018. The remaining six co-cited documents within the top ten list were all clinical trials pertaining to PARPis. Mirza et al. (2016) conducted a randomized, double-blind, phase III trial, designated as NOVA, to assess the efficacy of niraparib as a maintenance treatment for women with ROC. Their findings demonstrated that niraparib significantly prolonged the progression-free survival (PFS) duration (Mirza et al., 2016). Their related paper has rapidly garnered widespread attention, with the highest centrality in Table 6. The *New England Journal of Medicine* published an editorial asserting that PARP inhibitors possess the potential to revolutionize OC therapy in a unprecedented manner base on the results from the NOVA study (Spriggs and Longo, 2016). Subsequently, the SOLO-2 and ARIEL-3 studies demonstrated that both olaparib and rucaparib significantly improved progression-free survival (PFS) in patients with relapsed ovarian cancer, particularly among those harboring BRCA mutations (Pujade-Lauraine et al., 2017; Coleman et al., 2017). Articles from these two clinical trials soon achieved global recognition as well, and were ranked third and fifth among the top ten co-cited references, respectively. Given the promising outcomes observed in ROC, researchers have proceeded to incorporate PARPis into first-line therapy regimens. The second and sixth most popular references in Table 6 confirmed that patients with newly diagnosed advanced ovarian cancer could also benefit from olaparib and rucaparib (Moore et al., 2018; Gonzalez-Martin et al., 2019). Besides, there has been an increasing scholarly interest in the synergistic application of AIs and PARPis. Based on the findings of the PAOLA-1 study (Ray-Coquard et al., 2019), which was listed as seventh most cited reference in our analysis, the United States FDA approved the combination of olaparib and bevacizumab as a maintenance therapy for OC in 2022.

#### 4.2.4 Analysis of the co-cited keywords

Through the application of co-occurrence analysis, it is possible to systematically cluster the keywords within the research domain, thereby enabling the observation of the evolution of research trends, identification of prominent research hotspots, and elucidation of the interconnections among various keywords.

##### 4.2.4.1 The platinum-based chemotherapy

The entry “cisplatin” in the yellow cluster and “carboplatin” in the blue cluster, as depicted in Figure 8A, indicates the significance of platinum-based combination chemotherapy in the management of ROC. Based on the duration of the platinum-free interval (PFI), patients with ROC can be categorized into two groups: the PSR and the platinum-resistant recurrence (PRR). The Gynecologic Cancer InterGroup (GCIG) has stated that patients with OC who exhibit PSR are eligible for re-treatment with platinum-based agents (Friedlander et al., 2011). The first-generation platinum-based chemotherapeutic agent, cisplatin, has established the foundational framework for OC chemotherapy since its approval by the United States FDA in 1978. Carboplatin is closely related to cisplatin. However, carboplatin, the newer of the two, was somewhat less toxic than cisplatin, and has been used increasingly as the front-line agent in clinical practice (Markman, 1994). Oxaliplatin, the third-generation platinum agent, was primarily utilized based on the prior clinical experience due to lack of large-scale clinical trials for OC. Single agents generally yield partial responses; consequently, it has become standard practice to administer multiple agents in combination. Initially, the preferred regimen consisted of either cisplatin or carboplatin along with alkylating agents (Markman, 1994), like cyclophosphamide, ifosfamide and melphalan in the yellow cluster. At present, the combination of platinum with paclitaxel, gemcitabine, or doxorubicin is recommended as the standard chemotherapy regimen for PSR (Baert et al., 2021).

OC patients with PRR have a poor prognosis due to few treatment options with limited efficacy. In the past, it was reasonable to try hormonal therapy, as shown in the purple cluster, when platinum-free chemotherapy would have a limited chance of success and a high likelihood of toxicity; however, the overall response rate is less than 15% (Markman, 1994). Fortunately, the advent of the era of targeted therapy in OC offers renewed hope to individuals affected by PRR OC.

##### 4.2.4.2 The targeted therapy

Nowadays, researchers are increasingly realizing that management of ovarian cancer should be personalized based on the characteristic of the patient. For one thing, systematic lymphadenectomy is not recommended for women with ovarian cancer in early stage, especially for those affected by mucinous and low-grade serous histological subtype (Benedetti Panici et al., 2020). Accumulating evidences showed no survival benefits of lymphadenectomy among early-stage ovarian cancer patients. A multi-center randomized trial assessing the value of systematic lymphadenectomy in early ovarian cancer has revealed that there was no statistically significant difference in 5-year overall survival rates (84.0% versus 81.6%) between the lymphadenectomy group and the control group (Maggioni et al., 2006). Low-grade serous ovarian carcinoma exhibits a unique genetic profile characterized by *KRAS*/*BRAF* mutations compared to high-grade serous carcinoma, thus MEK inhibitors might be appropriate for the treatment of this malignancy (Perrone et al., 2024). For another, a comprehensive assessment is recommended prior to the management of an elderly person with ovarian cancer (Liontos et al., 2021). Elderly patients who are in good performance status should receive standard therapy identical to that of younger patients; In vulnerable elderly patients, the benefit/risk balance of surgery should be assessed, and various adapted chemotherapy modalities could be alternatives (Falandry and Gouy, 2019). Fader et al. compared the toxicities and outcomes of elderly ovarian cancer patients treated with standard-dose (carboplatin AUC 5-6 and paclitaxel 175 mg/m^2^) versus reduced-dose chemotherapy (carboplatin AUC 4-5 and paclitaxel 135 mg/m^2^), and found that reduced-dose carboplatin/paclitaxel may be better tolerated but equally effective as the standard regimen in elderly ovarian cancer patients (Fader et al., 2008). Similarly, the choice of the appropriate treatment regimen for ROC should also be decided on a case-by-case basis. Platinum-free interval, as a predictor of response to subsequent platinum re-treatment, has long been considered an essential factor to define treatment of recurrent ovarian cancer (Bergamini et al., 2019). Besides, the willingness for further therapy, age, general condition, comorbidities, extent and site of recurrent disease, and residual toxicity from previous treatments must be taken into consideration as well (Glajzer et al., 2020). Moreover, the availability of novel targeted therapies provides increased opportunities for implementing precision medicine in individuals with ROC.

Targeted therapy is characterized by the application of small-molecule drugs or monoclonal antibodies that specifically interact with molecules present on tumor cells or within their micro-environment to block cancer growth or spread, such as AIs, immune checkpoint inhibitors (ICIs) and PARPis.

Due to the strong correlation between vascular endothelial growth factor (VEGF) and OC, studies investigating the effects of AIs have been undertaken. In 2005, a 60-year-old woman with advanced, recurrent and refractory serous carcinoma of ovary firstly received the intravenous infusion of bevacizumab, and benefited an objective durable response lasting at least 5 months (Monk et al., 2005). Soon afterwards, the AURELIA study demonstrated that bevacizumab significantly increased the antitumor efficacy of paclitaxel in OC patients with PRR (Pujade-Lauraine et al., 2014). For those with PRS, bevacizumab combined with chemotherapy also significantly improved their objective response rate (ORR) and PFS from the data in the OCEANS study (Aghajanian et al., 2012). Therefore, bevacizumab has become the only AI approved for ROC treatment by the United States FDA, explaining its central position in our clustering analysis.

In contrast, ICIs now have limited efficacy for OC. Matulonis et al. (2019) concluded that single-agent pembrolizumab, a drug targeting the programmed cell death 1 (PD-1) receptor, showed modest activity in patients with ROC base on the KEYNOTE-100 study. However, our results showed that “immunotherapy” was still presented as a research hotspot in the co-cited keywords, indicating novel immunotherapeutic strategies for ovarian cancer are still an ongoing exploration in the clinical practice. Indeed, Drew et al. (2024) have declared that olaparib combined with durvalumab [a selective monoclonal antibody blocking programmed death-ligand 1 (PD-L1)] showed notable clinical activity in ovarian cancer patients with PSR in 2024. Therefore, combination therapies, particularly with PARPis, might be one of the future directions to enhance the benefit of immunotherapy. Further investigation is necessary to explore the selection of new ICI targets as well as non-immune targets. For the latter one, adoptive cell therapy might be an effective approach. Several clinical trials are currently ongoing in order to investigate the therapeutic efficacy of chimeric antigen receptor (CAR)-T cells targeting MUC16, mesothelin and folate receptor α on patients with ROC (Yang et al., 2020).

The introduction of PARPis has significantly transformed the landscape and paradigm of OC treatment. Clinical trials involving PARPis have made breakthroughs in the field of ROC maintenance treatment as well, holding great promise for the individuals previously considered incurable. The SOLO2 study evaluated olaparib tablet maintenance treatment in platinum-sensitive, relapsed OC patients with a germline *BRCA* (*gBRCA*) mutation who had received at least two lines of previous chemotherapy, and demonstrated that olaparib provided a significant PFS and overall-free survival (OS) improvement with no detrimental effect on quality of life (Pujade-Lauraine et al., 2017; Poveda et al., 2021). The OPINION analysis further confirmed that ROC patients without a *gBRCA* mutation also could gain clinical benefits from olaparib maintenance treatment (Poveda et al., 2022). Data from the NOVA trial revealed that patients with platinum-sensitive, recurrent ovarian cancer who were treated with niraparib experienced a significantly longer median progression-free survival (PFS) duration (Mirza et al., 2016). Researchers from China found that fuzuloparib maintenance therapy conferred a statistically significant and clinically meaningful improvement in PFS for patients with platinum-sensitive, recurrent ovarian cancer, regardless of g*BRCA 1/2* mutation base on the FZOCUS-2 study (Li et al., 2022). The keywords from aforementioned studies have been incorporated into our findings and visually represented through cluster analysis and timeline, emphasizing the current focus on PARPi and its significance in the realm of ROC therapy. Our findings have implications for clinical practice in the treatment of ROC. Wang et al. (2023) have confirmed that clinical application of PARPi as a maintenance therapy in Chinese patients with ROC was also effective in real world. In the future, research around the applications of PARPis in different scenarios for ROC treatment will be conducted. Recent research has demonstrated that surgery followed by maintenance treatment with PARP inhibitors may offer benefits in cases of recurrent ovarian cancer (Giannini et al., 2023); the NEO trial was performed to evaluate the pharmacodynamic effects of olaparib given prior to surgery for OC patients with PSR (2024 ASCO annual meeting, abstract No.: 5506). Moreover, PARPi resistance has become a problem that cannot be ignored. More than 40% of OC patients with *BRCA* mutation failed to benefit from PARPi, and 25%–50% of patients treated with PARPi will relapse (Li et al., 2020; Giannini et al., 2023). To enhance PARPi sensitivity, the optimal combination of PARPi and other treatment agents, such as oncolytic herpes simplex viruses (oHSVs), cyclin dependent kinases (CDK) inhibitors, ICIs and other DNA damage response-modifying drugs, should be considered (Li et al., 2020; Giannini et al., 2023).

##### 4.2.4.3 Combination of platinum-based chemotherapy and targeted therapy

We observed the evolution of ROC treatment from platinum-based chemotherapy to targeted therapy (Figure 8C). Meanwhile, traditional chemotherapeutic agents like cisplatin, carboplatin and paclitaxel still emerged as high-frequency words as shown in Figure 8B. We also found the constant linkages between traditional chemotherapy and targeted therapy from Figure 8D. In 2022, the United States FDA has withdrawn the approval of olaparib and rucaparib as the mono-therapeutic agents for ROC patients who have been treated with three or more prior lines of chemotherapy (Lee et al., 2023). The action indicated that benefits from PARPis should be built on the response to the platinum-based chemotherapy, which was in line with our results. Hence, PARPi were authorized for maintenance therapy with the goal of extending the benefits associated with chemotherapy, possibly enhancing PFS and OS rates, while ensuring minimal impact on quality of life of patients (Giannini et al., 2023).

### 4.3 Strengths and limitations of the study

The study utilized software tools, including Citespace and VOSviewer, to quantitatively and visually illustrate the findings, providing a more comprehensive portrayal of the research themes and trends in the field of ROC treatment. We gathered significant clinical trials pertaining to the treatment decision-making process for ROC and shared the latest research findings in the oral presentations at the 2024 ASCO annual conference. In contrast to the study conducted by Liu et al. (2023), our bibliometric analysis not only included the therapeutic strategies for OC patients with PRR, but scrutinized the treatment advancements for those with PSR.

This study surely has several limitations. Firstly, we limited our search to the MEDLINE database and the WOSCC, potentially resulting in the omission of certain articles. Fortunately, our study was deemed adequate for summarizing the research on ROC treatment due to the inclusion of over 10,000 articles. Secondly, lack of literature screen might lead to information redundancy. To mitigate potential shortcomings, extraneous terms were removed prior to conducting program analysis using the Citespace and VOSviewer software. Thirdly, bibliometric tools based on machine learning and natural language processing have the potential to introduce inherent system errors. For instance, it is not uncommon for multiple authors, particularly those of Chinese descent, to have identical names, which might result in the discrepancy of the data of the authors’ publications.

## 5 Conclusion

In summary, the bibliometric analysis revealed a consistent annual increase in the quantity of scholarly articles pertaining to the ROC treatment worldwide, beginning in 1990. Researchers from developed nations such as the United States and Italy, as well as developing countries like China took active part in advancing research on the treatment of ROC. Prominent international journals and professional periodicals focusing on gynecologic cancer have served as primary sources for the latest advancements and trends in the field by publishing large-scale clinical trials. Maintenance therapy using AIs or (and) PARPis has emerged as a significant complement to platinum-based chemotherapy for patients with ROC.

## Data Availability

The raw data supporting the conclusions of this article will be made available by the authors, without undue reservation.

## Abbreviations

OC, ovarian cancer; ROC, recurrent OC; WOSCC, web of science core collection; AIs, angiogenesis inhibitors; PARPis, poly (ADP-ribose) polymerase inhibitors; FDA, food and drug administration; PFS, progression-free survival; IF, impact factor; PFI, platinum-free interval; PSR, platinum-sensitive recurrent; PRR, platinum-resistant recurrent; ICIs, immune checkpoint inhibitors; VEGF, vascular endothelial growth factor; PD-1, programmed cell death 1; *gBRCA*, germline *BRCA*; OS, overall-free survival; HRD, homologous recombination deficiency.
